# Noisy Galvanic Stimulation Improves Roll-Tilt Vestibular Perception in Healthy Subjects

**DOI:** 10.3389/fneur.2018.00083

**Published:** 2018-03-01

**Authors:** Aram Keywan, Max Wuehr, Cauchy Pradhan, Klaus Jahn

**Affiliations:** ^1^German Center for Vertigo and Balance Disorders, Munich University Hospital, Munich, Germany; ^2^Department of Neurology, Schön Klinik Bad Aibling, Bad Aibling, Germany

**Keywords:** vestibular motion perception, noisy galvanic stimulation, stochastic resonance, vertigo, balance control

## Abstract

It has recently been demonstrated that noisy galvanic vestibular stimulation (nGVS) delivered as imperceptible white noise can improve balance control *via* the induction of stochastic resonance. However, it is unclear whether these balance improvements are accompanied by simultaneous enhancement to vestibular motion perception. In this study, 15 healthy subjects performed 8 quiet-stance tasks on foam with eyes closed at 8 different nGVS amplitudes ranging from 0 mA (baseline) to 0.5 mA. The nGVS amplitude that improved balance performance most compared to baseline was assigned as the optimal nGVS amplitude. Optimal nGVS amplitudes could be determined for 13 out of 15 subjects, who were included in the subsequent experimental procedures. The effect of nGVS delivered at the determined optimal intensity on vestibular perceptual thresholds was examined using direction-recognition tasks on a motion platform, testing roll rotations at 0.2, 0.5, and 1.0 Hz, both with active and sham nGVS stimulations. nGVS significantly reduced direction-recognition thresholds compared to the sham condition at 0.5 and 1.0 Hz, while no significant effect of nGVS was found at 0.2 Hz. Interestingly, no correlation was found between nGVS-induced improvements in balance control and vestibular motion perception at 0.5 and 1 Hz, which may suggest different mechanisms by which nGVS affects both modalities. For the first time, we show that nGVS can enhance roll vestibular motion perception. The outcomes of this study are likely to be relevant for the potential therapeutic use of nGVS in patients with balance problems.

## Introduction

It is commonly thought that the presence of noise in sensory systems has detrimental effects on the system’s ability to detect and process incoming signals. There is, however, growing evidence that under certain conditions an appropriate amount of noise can improve the signal-to-noise ratio in nonlinear systems and thereby enhance the recognition and transmission of the incoming information flow (1, 2). This phenomenon is based on a mechanism known as stochastic resonance (SR) in which the response of a nonlinear system to weak input signals can be optimized by the presence of a particular non-zero level of stochastic interference, i.e., noise (3). Dynamics consistent with this SR-mechanism have been demonstrated experimentally in human psychophysical studies on tactile sensation, auditory, and visual perception (4–6). Accordingly, external noise stimulation in these systems yields an improved processing of weak, sub-threshold stimuli, and thereby effectively lowers the system’s recognition threshold.

Recently, several studies examined the occurrence of SR-phenomena in the human vestibular system by means of galvanic vestibular stimulation (GVS). GVS is a technique to induce neural activity in vestibular afferents (semicircular canal and otolith afferents) and has been used to investigate vestibular functions for decades (e.g., vestibulo-spinal control of posture and locomotion; vestibulo-ocular control of eye movements) (7, 8). Using zero-mean white noisy GVS (nGVS) delivered at a low imperceptible intensity during static posturography, Iwasaki and colleagues observed a consistent improvement of body balance in healthy subjects as well as in patients with a bilateral vestibular hypofunction (BVH) (9, 10). Subsequently, nGVS was also found to effectively improve dynamic balance control during walking in healthy subjects and patients with BVH (11–13). Furthermore, nGVS was shown to enhance postural and motor performance in the elderly (14), as well as in patients with Parkinson’s disease (15, 16), and other neurodegenerative disorders (17). These beneficial effects of nGVS on static and dynamic body balance regulation were attributed to a noise-induced facilitation of vestibulo-spinal reflex function (18).

While there is now first evidence for nGVS-induced improvements in vestibular reflex functions, a possible parallel impact on the vestibulo-perceptual function remains to be determined. This could be particularly important for patients with BVH as they typically suffer from highly elevated perceptual thresholds in all motion planes (19). There is further evidence that human balance regulation in particular during unstable postural conditions not only requires accurate vestibulo-spinal reflex operation, but also significantly relies on vestibulo-perceptual capacities (20). Thus, the aim of this study was to examine whether nGVS effects on vestibulo-spinal function are accompanied by alterations in vestibulo-perceptual function. To this end, we (1) initially determined the individual nGVS intensity at which static balance performance of healthy participants improved optimally and (2) subsequently examined whether nGVS at the same intensity also affects vestibular perceptual function in a psychophysical direction-recognition task.

## Materials and Methods

Fifteen healthy subjects (seven females; mean age 25.1 ± 1.7 years) participated in the study. None of the participants reported any auditory, vestibular, neurologic, cardio-vascular, or other health impairments. All participants gave their written informed consent prior to the experiment. The study protocol was approved by the ethics committee of the medical faculty of the Ludwig-Maximilian University of Munich. The study was conducted in conformity with the Declaration of Helsinki.

### Galvanic Vestibular Stimulation

**Figure 1:**
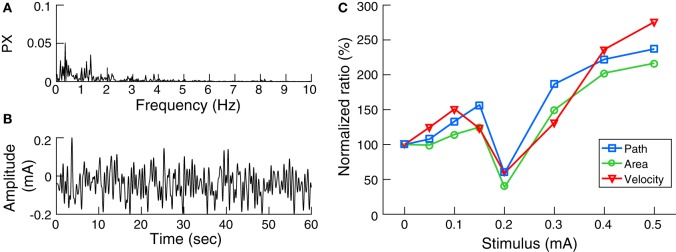
Noisy galvanic vestibular stimulation (nGVS) characteristics and effects on postural sway: **(A)** The power spectrum (PX) of head angular velocity in the medio-lateral axis of an individual subject. **(B)** Exemplary waveform of nGVS at 0.2 mA intensity. **(C)** The nGVS balance responses of a sample subject showing lower normalized ratios of the three postural parameters tested at 0.2 mA compared to baseline.

Galvanic vestibular stimulation was delivered through 4.0 cm × 6.4 cm electrodes (Axelgaard Manufacturing, Fallbrook, CA, USA) centered over the mastoid processes behind both ears. The skin surface was cleaned and dried and a layer of electrode gel was applied before electrode placement to achieve uniform current density and minimize any irritation to the skin due to stimulation. The impedance between the electrodes was confirmed to be less than 1 kΩ. Digital signals were generated using MATLAB and converted to analog signals *via* an NI USB-6221 data acquisition device (National Instruments, TX, USA). The analog command voltage signals were subsequently passed to a constant current stimulator (DS5, Digitimer, Hertfordshire, UK), which was connected to the stimulating electrodes. The stochastic signal consisted of zero-mean Gaussian white noise (nGVS) within a frequency range of 0–2 Hz (Figure 1B) (21). This bandwidth was chosen to cover the frequency range of head motion occurring during quiet stance as determined in two participants using a head fixed inertial sensor (EyeSeeCam, Munich, Germany) during standing with eyes closed on foam for 30 s (Figure 1A).

### Procedures

A common difficulty in interpreting results from SR studies is separating statistical variation from actual performance improvement at the optimal stimulus level. To avoid this issue, and due to the combined involvement of vestibulo-spinal and vestibulo-perceptual functions in maintaining upright posture (20), this study used a two-step experimental design, in which the optimal nGVS amplitude was first determined in a postural task and the same stimulus amplitude was then used for the vestibular motion perception tasks.

Initially, for each participant, the optimal nGVS intensity was determined during a 30 s stance trial on foam with eyes closed using a stabilometer platform (Kistler 9261 A, Kistler Group, Winterthur, Switzerland). Each participant performed eight stance trials with different nGVS peak amplitudes of 0 (i.e., baseline), 50, 100, 150, 200, 300, 400, and 500 µA presented in a pseudo-random order. Between trials, subjects had a 1 min break to alleviate any after-effects of the stimulation. Three body sway measures were recorded (9): the mean velocity of the center of pressure (COP) movement (i.e., the total distance traveled by the COP over time), the envelopment area traced by the movement of the COP, and the root mean square of the COP movement. Analysis of these parameters (except area, which is computed in 2D space) was carried out in the medio-lateral plane, since bipolar vestibular electrical stimulation has been shown to induce body sway primarily in this direction (22). The ratio of each parameter during the stimulation condition to that of the baseline condition was calculated. A reduction in the normalized values of these sway parameters indicates an improvement in postural control. The optimal nGVS intensity was then determined as the one at which balance measured during the stimulus condition was simultaneously smaller than that at baseline in at least two of the three COP parameters (Figure 1C).

After determining each participant’s optimal nGVS amplitude, subjects performed six direction-recognition experiments using a 6-degree of freedom motion platforms (Moog© 6DOF2000E, East Aurora, New York). Subjects were seated on a padded racing chair mounted on the motion platform. The head was rested on an inflatable padded pillow that adjusts itself to the actual head shape and was stabilized by placing large padded metal arms to fixate the subject’s head from both sides. These arms are an extension of larger 3-degree of freedom metal arms, which are firmly connected to the metal-bar structure supporting the chair of the platform. Noise-canceling headphones were then placed over the subjects’ ears to mask sound cues produced by the motion platform during the experiment. A two-buttoned (right and left) response box was handed to the subjects so that they could provide answers for the psychophysical task. Subjects’ eyes were covered by designated dark glasses to remove vision and all experiments were performed in darkness.

The vestibular perception thresholds of each participant were tested in the roll plane at three different frequencies: 0.2, 0.5, and 1.0 Hz, once with active nGVS stimulation and once with sham nGVS stimulation (i.e., electrodes and stimulator in place, but no stimulation delivered). The roll plane was specifically analyzed as literature has shown that galvanic stimulation produces sensation of rotation along this axis (23). The conditions were tested in a randomized order and participants were blinded to the stimulation protocol. Each experiment consisted of 150 trials, and thresholds were determined using the three-down one-up paradigm, which converges on the 79% correct threshold (24, 25). Each trial consisted of a single half-cycle that follows a raised-cosine profile to the right or to the left and subjects had to indicate the direction of movement by a button press (26, 27). A cumulative Gaussian distribution function was then fitted to the response data, which yielded a maximum likelihood psychometric fit (28). Similar to prior studies (29, 30), we have used a direction-recognition task to minimize the influence of vibration and other non-directional cues on vestibulo-perceptual thresholds.

### Outcome Measures

The primary outcome measure used in this study was the change in perceptual thresholds between the nGVS and sham conditions at the three frequencies tested. The secondary outcome analysis investigated possible correlations between improvements in the postural and perceptual performances.

### Statistical Analysis

Statistical analysis was performed on participants who showed an optimal nGVS response during the static posturography task. Descriptive statistics are presented as mean ± SD. Analysis of distribution of the recorded perceptual thresholds with the Kolmogorov–Smirnov test revealed significant departures from Gaussian distributions, which is in line with previous studies using comparable procedures (31, 32). However, none of the tested conditions revealed a significant departure from a normal distribution after velocity thresholds were expressed in logarithmic units, in accordance with previous studies (27, 29, 31, 32). Effects of nGVS on log-transformed motion perception thresholds were examined using a two-way repeated measures analysis of variance (ANOVA) with the factors condition (sham vs. nGVS) and frequency (0.2, 0.5, and 1 Hz) specified. Bonferroni *post hoc* analysis was employed to correct multiple testing. Pearson’s correlations were used to examine whether any significant relationship exists between the nGVS-induced improvements in balance performance and vestibular motion perception. Results were considered significant if *p* < 0.05. Statistical analysis was performed using SPSS (version 21.0, IBM Corp., USA).

## Results

For 13 out of 15 participants (six females, mean age = 25.7 ± 1.4 years), we found an optimal nGVS intensity at which static body balance effectively improved compared to the baseline trial. The two subjects who did not show this postural improvement could not be further subjected to the perceptual experiments. Table 1 presents the optimal nGVS levels determined for each of the 13 participants, together with the resultant effect on the three stance parameters analyzed.

**Table 1:** The optimal noisy galvanic vestibular stimulation (nGVS) amplitude of each subject and its effect on the three sway parameters in the medio-lateral plane are shown.

Subject	Optimal nGVS (μA)	Area (%)	Velocity (%)	Path (%)
1	100	−45.8	−8.1	−55.4
2	50	+10	−10.7	−17.4
3	150	−18.3	−3	−18.1
4	200	−50.3	−46.8	−42.7
5	100	−59.5	−12.9	−72.7
6	100	−7	−23.2	+3.7
7	50	−65.4	−32.7	+12.2
8	200	+4	−9.5	−67.8
9	150	−42.1	+18.8	−64.7
10	200	−22.7	+7.8	−34.4
11	100	−50.2	−28	+23
12	300	−72.3	−20.7	−11.5
13	50	−27.7	−34.1	+10.8
Mean	134.6 ± 86.3	−41.9 ± 20.6	−20.8 ± 13.4	−42.7 ± 23

*(−) signifies improvement and (+) signifies deterioration compared to baseline (0 µA)*.

**Figure 2:**
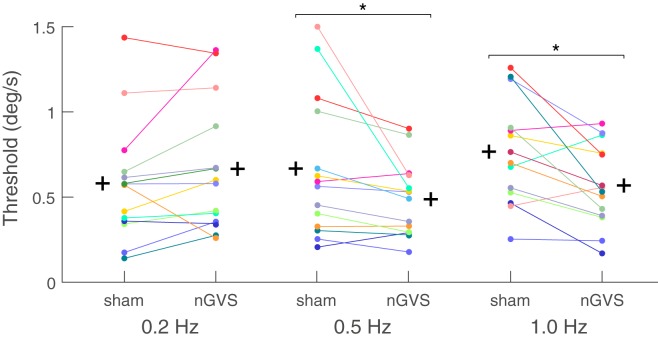
Motion recognition thresholds for the sham and noisy galvanic vestibular stimulation (nGVS) conditions for individual subjects: a significant nGVS-induced reduction in motion recognition thresholds was found at 0.5 and 1.0 Hz. nGVS did not affect motion recognition thresholds at 0.2 Hz. The (+) sign represents the group mean of each condition tested.

In the motion perception paradigm, the thresholds for the sham condition were in the range of previously published literature (29, 31). There was a significant main effect of nGVS on motion perception thresholds for the factor “condition” (i.e., sham vs. nGVS) (ANOVA, *F*_1,12_ = 7.406, *p* = 0.019), while no significant effect was found for the factor frequency (ANOVA, *F*_2,11_ = 1.323, *p* = 0.302). The interaction between the factors frequency and condition was, however, significant (ANOVA, *F*_2,11_ = 5.269, *p* = 0.020.). Analysis for individual frequencies revealed reduced motion perception thresholds in the nGVS condition compared to the sham condition for the 1 Hz roll motion (*F*_1,12_ = 8.455, *p* = 0.013; 0.56 vs. 0.76 deg/s, respectively; mean threshold reduction: 20.1 ± 0.5%). Similar results were also obtained for 0.5 Hz (*F*_1,12_ = 5.006, *p* = 0.045; 0.49 vs. 0.66 deg/s, respectively; mean threshold reduction: 14.1 ± 0.5%). For the 0.2 Hz condition only 3 out of 13 subjects showed a threshold reduction due to nGVS application (*F*_1,12_ = 1.408, *p* = 0.25, 0.70 vs.0.60 deg/s, respectively; mean threshold increase 9 ± 0.6%) (Figure 2).

**Figure 3:**
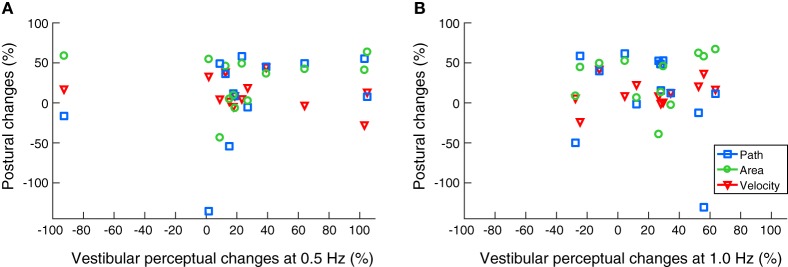
Scatter plots on noisy galvanic vestibular stimulation-induced improvements on body balance and vestibular motion perception. No significant correlations were found between the beneficial effects on posture and vestibular recognition thresholds at 0.5 Hz **(A)** and 1.0 Hz **(B)**.

No significant correlations were found between any of the improved body sway parameters and enhanced vestibular recognition thresholds at 0.5 and 1 Hz (Figure 3).

## Discussion

In this study, we show that nGVS not only improves stance performance in a static posturography paradigm (i.e., vestibulo-spinal function), but also influences vestibular perception in roll during a motion recognition task. Our results demonstrate that nGVS amplitudes, which enhance postural control, can also improve vestibular motion perception during roll rotations at 0.5 and 1.0 Hz, but not at 0.2 Hz. However, we did not observe any correlation between the nGVS-induced improvements during the static posturography task and their perceptual counterparts at 0.5 and 1.0 Hz.

Beneficial effects of nGVS on vestibular motion perception depended on the frequency of the roll-tilt stimulation, being effective at 0.5 and 1.0 Hz, but not at 0.2 Hz. Vestibulo-perceptual responses to roll-tilt stimulation have been characterized across a wide range of behaviorally relevant frequencies (29, 31–33). However, since roll-tilts activate both, the semicircular canals (SCCs) and otoliths, these studies do not provide answers concerning the relative contribution of these structures to the perception of roll tilts as a function of frequency. One such study has recently been published (34). It was found that motion perception thresholds for roll tilts at 0.5 and 1.0 Hz are predominantly determined by cues from the SCCs, while roll-tilt thresholds at 0.2 Hz include a substantial contribution from the otolith organs. This might explain the observed frequency dependence of nGVS on vestibular motion perception. Accordingly, the observed enhancements in vestibular perception at 0.5 and 1.0 Hz might predominantly reflect SR-enhanced signals from the SCCs. On the other hand, the presumed SR effect on roll-tilt perception at 0.2 Hz may be overridden by vector-cancelation taking place in the utricle during GVS stimulation (8).

Another outcome of our experiments was the apparent lack of correlation between improvements in the vestibulo-perceptual and vestibulo-spinal systems, both of which play an important role in the maintenance of upright postural stability (20). A possible contributing factor to this outcome could be the bandwidth of the stochastic vestibular stimulus we used in our study (0–2 Hz). Although this stimulation bandwidth has been previously validated to have high coherence with the frequencies governing postural sway responses in humans (21, 22), it did not show high coherence with responses of the lower limb and neck muscles (35, 36), both of which respond better at higher frequency bandwidths (0–20 and 0–70 Hz, respectively). Therefore, it could be possible that broader frequency bandwidths of stimulation, if also effective on vestibular perception, could, therefore, have more correlated outcomes with postural responses. Alternatively, the relative lack of correlation between nGVS effects on posture and perception may reflect a partial disassociation in processing vestibular cues along vestibulo-spinal and vestibular perceptual pathways; analogous to previous reports comparing vestibular cue processing between the vestibulo-ocular and vestibulo-perceptual systems (37, 38).

The outcomes we report in our study suggest that enhancements in balance control due to SR (11, 12). are likely to be accompanied by simultaneous perceptual improvements. Therefore, the potential implication for nGVS as a rehabilitation tool for patients with BVH could be paramount. This stems from the fact that patients with BVH suffer from highly elevated vestibulo-perceptual thresholds in all rotational and translational planes (19). Although nGVS improved vestibulo-spinal and vestibulo-perception differentially, the fact that both are actually enhanced by the same stimulation amplitude is highly important. This indicates that the same nGVS amplitude might be able to enhance both reflexive and perceptual performance of patients, regardless to the degree of enhancement it produces in each modality. Furthermore, both systems appear to be required to stabilize upright posture (20). Currently, the therapeutic regime in individuals with BVH is limited to physical therapy (39), where approximately only half of these patients benefit from this kind of intervention (40). The findings we report in this study, together with previous reports on nGVS-induced improvements in balance control as well as ocular-motor function (41) can promote an alternative or additional therapeutic option for reducing the postural imbalance and incidence of falls in this population.

Nevertheless, our study has some limitations. First, due to the lengthy testing time (4 h on average per participant), we chose only to investigate the effect of nGVS on vestibular perceptual performance in the roll plane, while not examining the other rotational and translational axes. Therefore, the improvements we show in this study may not necessarily hold true for other rotational and translational planes. Second, the frequency range for vestibular motion perception we tested was limited to the low-mid range, which may not fully encompass the frequency range of natural head motions during daily ambulation (around 0.5–5 Hz) (42). Third, our study had a relatively small sample size and the perceptual responses to stimulation exhibited by the study subjects were highly individual. This might be attributed to individual differences in inner ear anatomy, bone density, and possibly alteration in alertness to the perceptual task (although the latter is accounted for in the threshold calculation algorithm). Therefore, the current findings have to be confirmed in future on a larger study cohort.

In summary, we present here a first evidence for the sensitizing effect of nGVS on vestibular motion perception in healthy subjects. The results of this study could be a trigger to design therapeutic studies that use both the effects on balance control and on vestibular motion perception to improve mobility and quality of life in vestibular patients.

## Ethics Statement

The study protocol was approved by the ethics committee of the medical faculty of the Ludwig-Maximilian University of Munich. All subjects gave written informed consent in accordance with the Declaration of Helsinki.

## Author Contributions

AK: concept, design, programming experiments, data collection, data analysis, creating Figures 1–3, and writing of manuscript. MW: concept, design, Matlab codes for stance performance analysis, data analysis, editing Figures 1–3, and review of manuscript. CP: programming the nGVS stimulation paradigm, data analysis, and review of manuscript. KJ: concept, design, review, and amendment of manuscript. All authors have approved the final version of the manuscript and are agreed to be accountable for all aspects of the work.

## Conflict of Interest Statement

The authors declare that the research was conducted in the absence of any commercial or financial relationships that could be construed as a potential conflict of interest. The reviewer CM and handling Editor declared their shared affiliation.
